# Patient-Level and County-Level Trends in Nonfatal Opioid-Involved Overdose Emergency Medical Services Encounters — 491 Counties, United States, January 2018–March 2022

**DOI:** 10.15585/mmwr.mm7134a1

**Published:** 2022-08-26

**Authors:** Shannon M. Casillas, Cassandra M. Pickens, Erin K. Stokes, Josh Walters, Alana Vivolo-Kantor

**Affiliations:** ^1^Division of Overdose Prevention, National Center for Injury Prevention and Control, CDC; ^2^biospatial, Inc., Durham, North Carolina.

The number of nonfatal opioid-involved overdoses treated by health care providers has risen in the United States; the median number of emergency department (ED) visits for these overdoses was significantly higher during 2020 than during 2019 (*1*). ED visit data can underestimate nonfatal opioid-involved overdose incidence because, increasingly, persons experiencing a nonfatal opioid overdose are refusing transport to EDs by emergency medical services (EMS) (*2*). A study in Kentucky found that during a 6-month period, 19.8% of persons treated by EMS for an opioid overdose refused transport to an ED (*2*). Thus, EMS encounter data involving suspected nonfatal opioid-involved overdoses complement ED data and also allow for near real-time analysis (*3*). This report describes trends in rates of EMS encounters for nonfatal opioid-involved overdoses per 10,000 total EMS encounters (rates) by selected patient- and county-level characteristics during January 2018–March 2022 in 491 counties from 21 states using data from biospatial, Inc.* During this period, the nonfatal opioid-involved overdose rate increased, on average, 4.0% quarterly. Rates increased for both sexes and for most age groups. Rates were highest among non-Hispanic White (White) and non-Hispanic Native Hawaiian or other Pacific Islander (NH/OPI) persons, and increases were largest among non-Hispanic Black (Black), followed by Hispanic or Latino (Hispanic) persons. Rates increased in both urban and rural counties and for all quartiles of county-level characteristics (i.e., unemployment, education, and uninsured), except in counties with the lowest percentage of uninsured persons. Rates were highest and rate increases were largest in urban counties and counties with higher unemployment rates. This analysis of nonfatal opioid-involved overdose trends in EMS data highlights the utility of these data and the importance of addressing inequities that contribute to disproportionate overdose risk, such as through focused outreach to racial and ethnic minority groups, who disproportionately experience these inequities, and communities with higher levels of unemployment. EMS providers are in a unique position to engage in postoverdose response protocols and promote evidence-based overdose education and facilitate linkage to care and harm reduction services.^†^^,^^§^

EMS data collected by biospatial, Inc. from 491 counties in 21 states^¶^ with consistent data coverage** were analyzed by quarter during January 2018–March 2022. The Council of State and Territorial Epidemiologists (CSTE) standard guidance for querying EMS data for nonfatal opioid-involved overdoses was applied. The CSTE EMS Nonfatal Opioid Overdose Standard Guidance (published May 2022) queries coded data elements (provider’s primary and secondary impression, primary and other associated symptoms, medication administered, and response to medication) and a free text field (patient care report narrative) to identify suspected nonfatal opioid-involved overdose encounters.^††^ Encounters were included if the type of service requested was an emergency response and excluded if a fatal encounter was indicated, or if the encounter was cancelled or was an assist to the primary responding unit.^§§^

Trends were analyzed overall, by patient characteristics (i.e., age, sex, and race and ethnicity)^¶¶^; incident disposition (i.e., transported or not transported by EMS); percentage unemployed, percentage of population aged ≥25 years who are high school graduates or higher, and percentage uninsured (derived from the U.S. Census Bureau American Community Survey***); and the following county-level (incident location) characteristics: urban or rural classification.^†††^ Each American Community Survey variable was categorized into quartiles. The rate of nonfatal opioid-involved overdose EMS encounters per 10,000 total EMS encounters was calculated. Rates, rather than counts, were used to account for fluctuations in EMS use over time.

Joinpoint regression (version 4.9; National Cancer Institute) was used to measure the average quarterly percent change (AQPC) for the entire study period and quarterly percent change for each trend segment; the permutation model selection method was used, and the maximum number of joinpoints allowed was three. P<0.05 was considered statistically significant. This activity was reviewed by CDC and conducted consistent with applicable federal law and CDC policy.^§§§^

The rate of nonfatal opioid-involved overdose EMS encounters increased, on average, 4.0% per quarter during January 2018–March 2022, increasing from 98.1 per 10,000 EMS encounters during Quarter 1 (Q1)^¶¶¶^ 2018 to 179.1 during Q1 2022 (Table). Nonfatal opioid-involved overdose rates increased for most strata; the most common inflection points were Quarter 3 (Q3) 2019 and Quarter 2 (Q2) 2020 (Figure 1) (Figure 2). Beginning in Q3 2020, overall nonfatal opioid-involved overdose rates stabilized after the onset of the COVID-19 pandemic.

**TABLE:** Joinpoint regression analysis of trends* in rates of emergency medical services encounters for nonfatal opioid-involved overdoses, overall and by patient- and county-level characteristics, by quarter — 491 counties, United States, January 2018–March 2022

Characteristic	Average quarterly % change (95% CI)	No. of joinpoints	Trend segments, quarterly % change (95% CI)		
			Segment 1	Segment 2	Segment 3
**Overall**	4.0% (2.3 to 5.8)*	**1**	Q1 2018–Q3 2020 6.6 (4.6 to 8.6)*	**Q3 2020–Q1 2022 −0.1 (−4.1 to 4.0)**	**NA**
**Patient-level**					
**Age group, yrs**					
0–14	5.8 (4.0 to 7.6)*	0	NA	NA	NA
15–24	3.0 (−0.1 to 6.3)	2	Q1 2018–Q3 2019 0.8 (−2.1 to 3.7)	Q3 2019–Q2 2020 17.9 (−0.8 to 40.1)	Q2 2020–Q1 2022 −0.8% (−3.1 to 1.5)
25–34	3.3 (0.9 to 5.7)*	2	Q1 2018–Q3 2019 3.1 (0.9 to 5.5)*	Q3 2019–Q2 2020 12.9 (−1.0 to 28.8)	Q2 2020–Q1 2022 −0.5% (−2.3 to 1.2)
35–54	5.3 (4.1 to 6.6)*	1	Q1 2018–Q2 2020 8.6 (6.9 to 10.3)*	Q2 2020–Q1 2022 1.3 (−1.0 to 3.6)	NA
≥55	3.2 (2.3 to 4.1)*	0	NA	NA	NA
**Sex**					
Female	3.1 (2.2 to 4.1)*	0	NA	NA	NA
Male	4.7 (3.3 to 6.1)*	1	Q1 2018–Q2 2020 7.8 (5.9 to 9.7)*	Q2 2020–Q1 2022 0.9 (−1.7 to 3.5)	NA
**Race and ethnicity^†^**					
American Indian or Alaska Native	3.1 (0.7 to 5.7)*	1	Q1 2018–Q2 2019 −4.0 (−10.7 to 3.2)	Q2 2019–Q1 2022 6.5 (4.3 to 8.9)*	NA
Asian	5.2 (3.3 to 7.0)*	0	NA	NA	NA
Black or African American	7.4 (5.0 to 9.7)*	1	Q1 2018–Q2 2020 13.5 (10.3 to 16.7)*	Q2 2020–Q1 2022 0 (−4.1 to 4.3)	NA
Hispanic or Latino	5.7 (4.4 to 7.0)*	1	Q1 2018–Q2 2020 10.4 (8.6 to 12.2)*	Q2 2020–Q1 2022 0 (−2.4 to 2.3)	NA
Native Hawaiian or other Pacific Islander	0.9 (−1.4 to 3.2)	0	NA	NA	NA
White	3.4 (2.3 to 4.4)*	0	NA	NA	NA
**Disposition**					
Not transported by EMS	7.1 (3.5 to 10.7)*	2	Q1 2018–Q3 2019 3.9 (0.6 to 7.2)*	Q3 2019–Q2 2020 23.2 (2.0 to 48.8)*	Q2 2020–Q1 2022 3.5% (0.9 to 6.1)*
Transported by EMS	3.9 (2.0 to 5.9)*	1	Q1 2018–Q3 2020 6.6 (4.5 to 8.7)*	Q3 2020–Q1 2022 −0.4 (−4.6 to 4.0)	NA
**County-level**					
**Unemployment rate, % (quartile)^§^**					
0–3.6	2.1 (1.2 to 3.0)*	0	NA	NA	NA
3.7–4.9	3.1 (2.0 to 4.3)*	0	NA	NA	NA
5.0–6.3	4.0 (2.9 to 5.0)*	0	NA	NA	NA
6.4–30.4	5.9 (3.1 to 8.8)*	1	Q1 2018–Q2 2020 11.2 (7.4 to 15.2)*	Q2 2020–Q1 2022 −0.6 (−5.6 to 4.7)	NA
**High school graduate or higher, % (quartile)^§^**					
21.9–84.1	3.6 (2.6 to 4.6)*	0	NA	NA	NA
84.2–88.8	5.0 (2.9 to 7.1)*	1	Q1 2018–Q2 2020 10.0 (7.2 to 12.9)*	Q2 2020–Q1 2022 −1.1 (−4.8 to 2.7)	NA
88.9–92.1	4.3 (3.1 to 5.4)*	0	NA	NA	NA
92.2–98.6	3.1 (2.0 to 4.3)*	0	NA	NA	NA
**Uninsured, % (quartile)^§^**					
0–5.8	0.9 (−0.1 to 1.9)	0	NA	NA	NA
5.9–8.5	3.3 (2.2 to 4.4)*	0	NA	NA	NA
8.6–12.0	5.5 (3.3 to 7.7)*	1	Q1 2018–Q2 2020 10.2 (7.2 to 13.2)*	Q2 2020–Q1 2022 −0.3 (−4.1 to 3.7)	NA
12.1–42.6	3.6 (1.7 to 5.6)*	2	Q1 2018–Q2 2019 0.2 (−2.8 to 3.4)	Q2 2019–Q2 2020 14.3 (6.6 to 22.6)*	Q2 2020–Q1 2022 0.3% (−1.6 to 2.2)
**Urbanicity**					
Urban	4.2 (2.4 to 6.0)*	1	Q1 2018–Q3 2020 7.0 (5.0 to 9.0)*	Q3 2020–Q1 2022 −0.3 (−4.3 to 3.8)	NA
Rural	2.8 (1.9 to 3.7)*	0	NA	NA	NA

**Abbreviations:** EMS = emergency medical services; NA = not applicable; Q1 = quarter 1; Q2 = quarter 2; Q3 = quarter 3.

* P<0.05 was considered statistically significant.

^†^ Persons of Hispanic or Latino ethnicity, regardless of race, were classified as Hispanic. For the remaining categories, persons who were non-Hispanic are reported by their indicated single race classification (e.g., Asian, Black, or White). Persons with other, unknown, or missing race or ethnicity were excluded.

^§^ The cutoffs for each quartile (derived from the U.S. Census Bureau American Community Survey) are shown (e.g., the first unemployment rate quartile included counties with unemployment rates from 0 to 3.6%).

**FIGURE 1:**
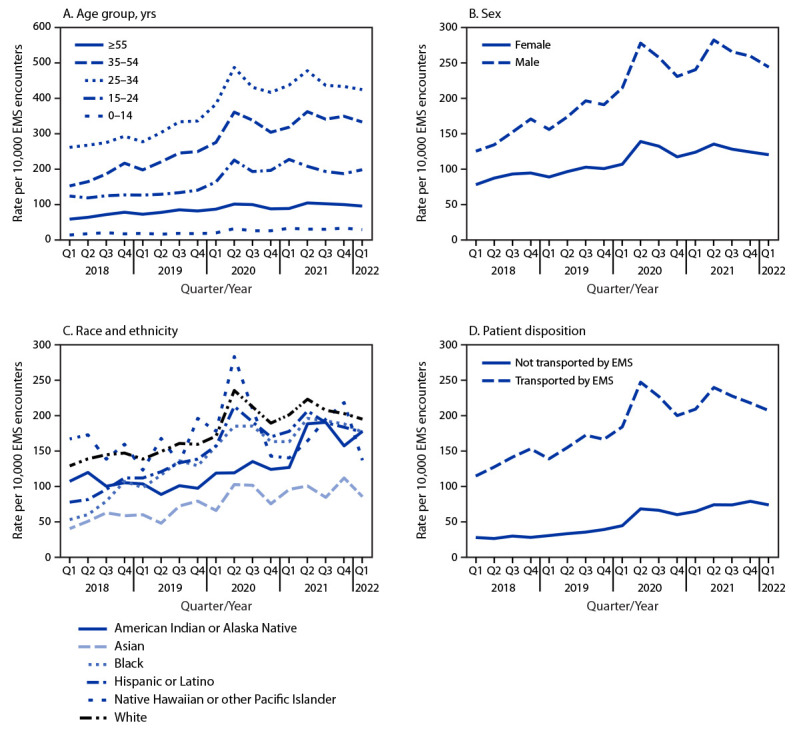
Nonfatal opioid-involved overdose rates by age group (A), sex (B), race and ethnicity (C),* and patient disposition (D), by quarter — 491 counties, United States, January 2018–March 2022 **Abbreviations:** EMS = emergency medical services; Q1 = quarter 1; Q2 = quarter 2; Q3 = quarter 3; Q4 = quarter 4. * Persons of Hispanic or Latino ethnicity, regardless of race, were classified as Hispanic. For the remaining categories, persons who were non-Hispanic are reported by their indicated single race classification (e.g., Asian, Black, or White). Persons with other, unknown, or missing race or ethnicity were excluded.

**FIGURE 2:**
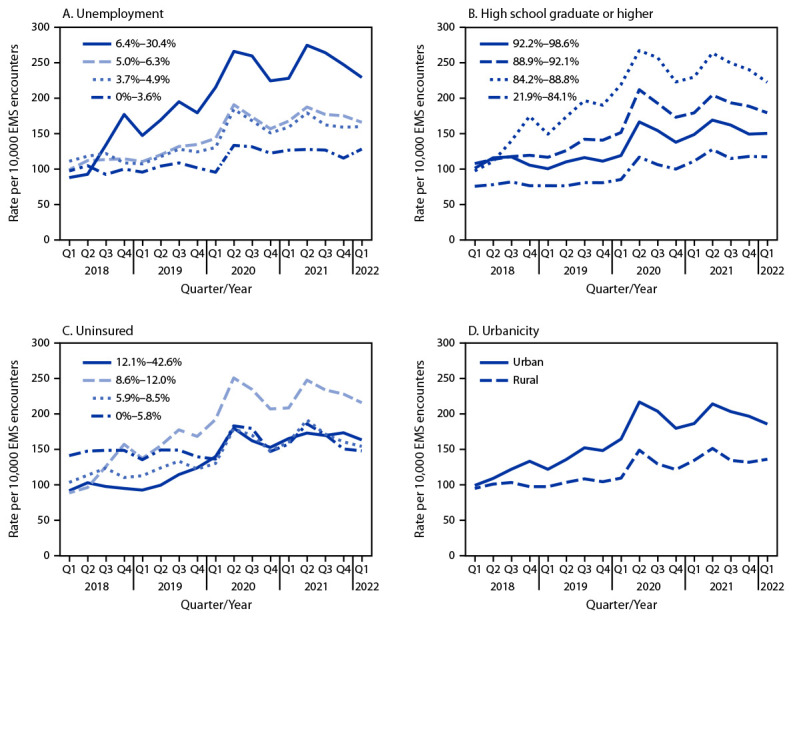
Nonfatal opioid-involved overdose rates by county-level unemployment (A), education (B), percentage uninsured (C),* and urbanicity (D), by quarter — 491 counties, United States, January 2018–March 2022 **Abbreviations:** EMS = emergency medical services; Q1 = quarter 1; Q2 = quarter 2; Q3 = quarter 3; Q4 = quarter 4. * County-level unemployment, education, and percentage uninsured were categorized into quartiles.

## Patient-Level Characteristics

Nonfatal opioid-involved overdose rates were highest among adults aged 25–34 years and lowest among children and adolescents aged 0–14 years (Figure 1). AQPCs for the entire study period were positive in all age groups except 15–24 years (range = 3.2%–5.8%). Rates were higher in males than in females, and the disparity widened over time (AQPC for males, 4.7%; for females, 3.1%) (Figure 1). Rates were highest among White and NH/OPI persons and lowest among non-Hispanic Asian (Asian) persons (Figure 1). Rates increased significantly among all racial and ethnic groups except NH/OPI persons; among groups with an increase, AQPCs ranged from 3.1% to 7.4%. Rates increased, on average, 3.4% quarterly among White persons, whereas increases were substantially higher among Black (7.4%) and Hispanic persons (5.7%). Rates were higher among persons transported by EMS than among those not transported by EMS (Figure 1); however, increases were larger among those not transported by EMS (AQPC for those not transported, 7.1%; for those transported, 3.9%).

## County-Level Characteristics

Nonfatal opioid-involved overdose rates were higher in counties with higher unemployment (Figure 2); rates increased faster in counties with higher unemployment, with the AQPC for the entire period ranging from 2.1% in counties in the lowest quartile of unemployment to 5.9% in counties in the highest quartile. Rates were lowest in counties with the smallest proportion of high school graduates and highest among counties with the next smallest proportion (Figure 2). The AQPC for the entire study period was positive for all education quartiles (range = 3.1%–5.0%). AQPCs were positive (range = 3.3%–5.5%) for all quartiles of uninsured except the lowest quartile; rate increases were largest for the third quartile, which had the lowest rate in Q1 2018 and the highest rate in Q1 2022, more than doubling from 88.1 to 215.8 per 10,000 EMS encounters (Figure 2). Rates were higher in urban than in rural counties, and the disparity increased over time (AQPC for urban counties, 4.2%; for rural counties, 2.8%) (Figure 2).

## Discussion

This report highlights several findings: 1) rates of nonfatal opioid-involved overdose EMS encounters per 10,000 total EMS encounters increased steadily from 2018 through the onset of the COVID-19 pandemic; 2) nonfatal opioid-involved overdose rates increased for both sexes, all age groups except persons aged 15–24 years, and all racial and ethnic groups except NH/OPI; 3) nonfatal opioid-involved overdose rates increased among all quartiles of county-level characteristics, except for counties with the lowest percentage of uninsured persons; and 4) higher nonfatal opioid-involved overdose rates and rate increases were observed in urban counties and in counties with higher unemployment rates.

Increases in nonfatal opioid-involved overdose EMS encounters through Q3 2020 are consistent with increases in nonfatal opioid-involved overdoses treated in EDs (*1*) and synthetic opioid-involved overdose deaths.**** Nonfatal opioid-involved overdose rates in this study remained stable during Q3 2020–Q1 2022, which is consistent with opioid-involved overdose ED visits in CDC’s Drug Overdose Surveillance and Epidemiology system.^††††^ However, this finding is unlike those for mortality data, which have demonstrated increases in opioid-involved overdose deaths during this period. Further exploration into the types of opioids (e.g., fentanyl, heroin, and prescribed opioids) contributing to overdoses and the shifting drug supply will assist in better interpretation of these differences.

The increase in nonfatal opioid-involved overdose rates for most demographic groups is similar to findings from ED data (*4*). Although rates were highest among White and NH/OPI persons, rate increases were largest among Black, followed by Hispanic persons. According to a recent study, Black persons experienced the largest increase in fatal all-drug overdoses during 2019–2020 (*5*). Structural barriers, mistrust in the health care system, and other disparities that contribute to overdose risk underscore the need to address inequities, particularly among minority populations, as part of a comprehensive response to the U.S. drug overdose crisis (*5*).

This report highlights community characteristics that are associated with higher nonfatal opioid-involved overdose rates, such as county-level unemployment. This finding is consistent with a systematic review that reported that recessions and unemployment increased psychological stress and subsequent illegal drug use (*6*,*7*). Counties with the lowest percentage of uninsured persons represented the only quartile without a significant increase in the rate of nonfatal opioid-involved overdoses. A previous study found that drug overdose mortality was elevated in U.S. Census Bureau tracts with higher rates of uninsured persons (*8*); however, in the current analysis, the quartile with the second highest percentage of uninsured persons had the highest rate and largest overall rate increase in nonfatal opioid-involved overdoses. Persons who are uninsured might be less likely to use EMS after an overdose; a study in Wisconsin found that Medicaid expansion resulted in an increase in the share of opioid-related ED visits covered by Medicaid among men aged 19–49 and women aged 19–29 years (*9*). In contrast to previous research reporting a higher rate of nonfatal opioid-involved overdose ED discharges in rural areas with lower levels of educational attainment (*10*), rates in the current analysis were lowest in counties with the smallest proportions of high school graduates. This divergent finding might be because of moderation by urbanicity or differences between ED discharge and EMS data (*10*).

The findings in this report are subject to at least five limitations. First, analyses are not nationally representative; therefore, the results cannot be generalized. Second, there are no toxicology results in EMS records to confirm the substance involved in suspected overdoses; however, EMS providers are trained to recognize the signs and symptoms of an opioid overdose so that they can administer appropriate treatment.^§§§§^ Third, analyses were not able to identify reasons a person might or might not have been transported by EMS after an encounter. It is possible persons who were transported were more likely to be in critical condition (e.g., unconscious) compared with those not transported, and nontransport could have been because of factors other than refusal (e.g., hospitals were at capacity). Fourth, despite only including counties with consistent data coverage, during the onset of the COVID-19 pandemic in March 2020, total EMS encounters decreased by 12.6% in Q2 2020 compared with the previous quarter, and nonfatal opioid-involved EMS encounters increased 15.2%; thus, nonfatal opioid-involved overdose rates might be inflated during this time. Finally, quality and completeness of EMS data might vary by period, reporting agency, and location.

These findings illustrate the utility of EMS data to monitor nonfatal opioid-involved overdose trends, especially given past research findings indicating that persons are increasingly refusing EMS transport to EDs after an overdose (*2*). A study in Kentucky found that during January 14‒April 26, 2020, 19.8% of patients treated by EMS for an opioid overdose refused transport to an ED, increasing from 16.4% before the onset of the COVID-19 pandemic to 22.4% after the onset (*2*). This analysis of nonfatal opioid-involved overdose trends highlights the need for increased access to services (e.g., harm reduction) among all populations, and also identifies characteristics of communities that are disproportionately affected by overdoses, such as those with higher unemployment rates. These data can guide public health efforts to ensure implementation of equitable prevention and response initiatives; for example, counties with higher unemployment rates might benefit from increased access to harm reduction services (e.g., naloxone and fentanyl test strip distribution), treatment (e.g., medications for opioid use disorder^¶¶¶¶^), and behavioral health services. Systems of care, which include EMS, mobile-integrated health, and community paramedicine, could collectively deploy to improve access to treatment and promote harm reduction strategies. For example, the Studying the PhilAdelphia Resilience Project as a Response to Overdose (SPARRow) program has staff members who accompany ambulances responding to overdoses and deliver harm reduction and care linkage to persons who refuse hospital transport.***** EMS data can also improve understanding of prehospital trends in nonfatal opioid-involved overdoses in near real time to guide tailored public health response and prevention efforts.

**Summary**

**What is already known about this topic?**

Nonfatal opioid-involved overdoses treated in emergency departments (EDs) are increasing, yet ED surveillance does not capture all overdoses because persons who had a nonfatal opioid-involved overdose often refuse transport by emergency medical services (EMS).

**What is added by this report?**

The rate of nonfatal opioid-involved overdose EMS encounters increased, on average, 4.0% quarterly during January 2018–March 2022, from 98.1 to 179.1 per 10,000 EMS encounters. Rates increased across most sociodemographic and county characteristics.

**What are the implications for public health practice?**

Monitoring nonfatal opioid-involved overdose trends in EMS data in near real time can help identify communities disproportionately affected by overdose and can guide equitable response and prevention efforts, including increased access to harm reduction services and linkage to care and treatment.

## Footnotes

* biospatial, Inc. receives EMS data from 43 states; among these states, 25 are full coverage and 18 are partial. For full coverage states, biospatial receives all records that the state office receives; for partial coverage states, biospatial receives some of the data from sources other than the state office (e.g., through partnerships directly with EMS providers). https://www.biospatial.io/

†
https://emergency.cdc.gov/han/2020/han00438.asp

§
https://www.cdc.gov/drugoverdose/pdf/pubs/2018-evidence-based-strategies.pdf

¶ The following states that share data with biospatial, Inc. included one or more counties that met the underlying event coverage (UEC) threshold (≥75%) for sufficient coverage during the study period and were included in the analysis: Alabama (17 counties), Alaska (two), Arizona (one), Arkansas (35), California (two), Colorado (three), Florida (32), Georgia (92), Illinois (49), Kansas (64), Michigan (29), Montana (13), New Mexico (14), Oregon (one), Rhode Island (five), South Carolina (41), Texas (three), Virginia (62), Washington (one), Wisconsin (seven), and Wyoming (18).

** UEC is a ratio of the records received by biospatial, Inc. compared with the estimated number of all EMS encounters expected for the specified geographic area (e.g., county); this metric is calculated using probabilistic models of historic data and county population characteristics. For this analysis, records from counties that had UEC ≥75% for each quarter during the study period were eligible for inclusion.

††
https://cdn.ymaws.com/www.cste.org/resource/resmgr/opioidsurv/EMS_Nonfatal_Opioid_Overdose.pdf

§§ Encounters were excluded if initial or final patient acuity or incident patient disposition indicated a fatal encounter or if incident patient disposition indicated the encounter was cancelled or that the encounter was an assist to the primary responding unit.

¶¶ Persons of Hispanic or Latino ethnicity, regardless of race, were classified as Hispanic. For the remaining categories, persons who were non-Hispanic were reported by their indicated single race classification (e.g., Asian, Black, or White). Persons with other, unknown, or missing race or ethnicity were excluded.

*** The U.S. Census Bureau American Community Survey is conducted nationwide and collects information from a representative sample of households to understand demographic, social, economic, and housing characteristics at the county level. For this analysis, the 2020 American Community Survey 5-year estimates were queried. https://www.census.gov/data/developers/data-sets/acs-5year.html

††† County urbanization was categorized using 2013 National Center for Health Statistics data. Urban counties were those considered to be large central metropolitan, large fringe metropolitan, medium metropolitan, and small metropolitan; rural counties were those considered to be micropolitan or noncore. https://www.cdc.gov/nchs/data_access/urban_rural.htm

§§§ 45 C.F.R. part 46.102(l)(2), 21 C.F.R. part 56; 42 U.S.C. Sect. 241(d); 5 U.S.C. Sect. 552a; 44 U.S.C. Sect. 3501 et seq.

¶¶¶ Quarters were defined as Q1 (January 1–March 31), Q2 (April 1–June 30), Q3 (July 1–September 30), and Q4 (October 1–December 31).

****
https://www.cdc.gov/nchs/nvss/vsrr/drug-overdose-data.htm

††††
https://www.cdc.gov/drugoverdose/nonfatal/dashboard/index.html

§§§§
https://www.ems.gov/projects/opioid-crisis.html

¶¶¶¶
https://www.cdcfoundation.org/sites/default/files/files/PHAST_Web_Toolkit_Pilot_Version_2.0_For_Dissemination.pdf

*****
https://app.dimensions.ai/details/grant/grant.8632499
